# Urinary Vitamin D Binding Protein and KIM-1 Are Potent New Biomarkers of Major Adverse Renal Events in Patients Undergoing Coronary Angiography

**DOI:** 10.1371/journal.pone.0145723

**Published:** 2016-01-11

**Authors:** Lyubov Chaykovska, Fabian Heunisch, Gina von Einem, Markus L. Alter, Carl-Friedrich Hocher, Oleg Tsuprykov, Thomas Dschietzig, Axel Kretschmer, Berthold Hocher

**Affiliations:** 1 Center for Cardiovascular Research, Charité Universitaetsmedizin Berlin, Berlin, Germany; 2 Department of Vascular Surgery, University Hospital Zurich, Zurich, Switzerland; 3 Institute for Nutritional Science, University of Potsdam, Potsdam, Germany; 4 Immundiagnostik AG, Bensheim, Germany; 5 Department of Cardiology and Angiology, Charité Universitaetsmedizin Berlin, Berlin, Germany; 6 Bayer Pharma AG, Wuppertal, Germany; 7 IFLb Laboratoriumsmedizin Berlin GmbH, Berlin, Germany; The University of Manchester, UNITED KINGDOM

## Abstract

**Background:**

Vitamin-D-binding protein (VDBP) is a low molecular weight protein that is filtered through the glomerulus as a 25-(OH) vitamin D 3/VDBP complex. In the normal kidney VDBP is reabsorbed and catabolized by proximal tubule epithelial cells reducing the urinary excretion to trace amounts. Acute tubular injury is expected to result in urinary VDBP loss. The purpose of our study was to explore the potential role of urinary VDBP as a biomarker of an acute renal damage.

**Method:**

We included 314 patients with diabetes mellitus or mild renal impairment undergoing coronary angiography and collected blood and urine before and 24 hours after the CM application. Patients were followed for 90 days for the composite endpoint major adverse renal events (MARE: need for dialysis, doubling of serum creatinine after 90 days, unplanned emergency rehospitalization or death).

**Results:**

Increased urine VDBP concentration 24 hours after contrast media exposure was predictive for dialysis need (no dialysis: 113.06 ± 299.61ng/ml, n = 303; need for dialysis: 613.07 ± 700.45 ng/ml, n = 11, Mean ± SD, p<0.001), death (no death during follow-up: 121.41 ± 324.45 ng/ml, n = 306; death during follow-up: 522.01 ± 521.86 ng/ml, n = 8; Mean ± SD, p<0.003) and MARE (no MARE: 112.08 ± 302.00ng/ml, n = 298; MARE: 506.16 ± 624.61 ng/ml, n = 16, Mean ± SD, p<0.001) during the follow-up of 90 days after contrast media exposure. Correction of urine VDBP concentrations for creatinine excretion confirmed its predictive value and was consistent with increased levels of urinary Kidney Injury Molecule-1 (KIM-1) and baseline plasma creatinine in patients with above mentioned complications. The impact of urinary VDBP and KIM-1 on MARE was independent of known CIN risk factors such as anemia, preexisting renal failure, preexisting heart failure, and diabetes.

**Conclusions:**

Urinary VDBP is a promising novel biomarker of major contrast induced nephropathy-associated events 90 days after contrast media exposure.

## Introduction

Acute kidney injury (AKI) is a severe condition associated with a mortality of up to to 70% [1,2,3,4,5] and prolonged hospitalitation increasing the costs of treatment [6].

**Fig 1:**
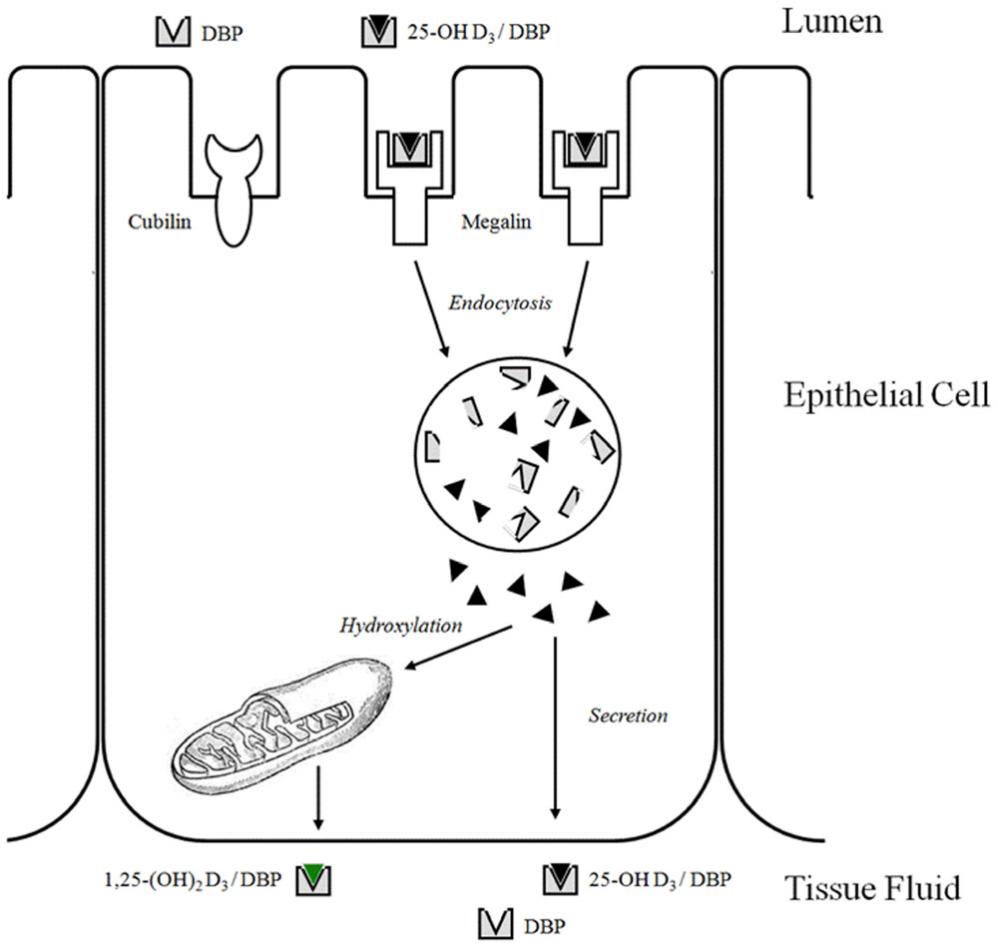
Model of megalin function in renal uptake and activation of 25-(OH) Vitamin D3. [11]

Biomarkers that can predict the disease progression or development of life threatening complications may help to better understand the disease pathways and to prevent complications. During AKI, increased urinary excretion of different molecules may be caused by either increased tubular secretion or impaired proximal tubular reabsorption [7,8,9]. Some biomarkers such as liver-type fatty acid-binding protein (L-FABP) and neutrophil gelatinase-associated lipocalin (NGAL) are secreted by normal tubular cells into the urine, however, increased amounts of those biomarkers in plasma of AKI patients [10] may also increase their urinary concentration, causing misinterpretation. Vitamin-D-binding protein (VDBP) is a low molecular weight protein that is filtered through the glomerulus as a 25-(OH) vitamin D 3/VDBP complex and is uptaken by megalin receptors in the brush border of proximal tubule cells. The carrier VDBP is degraded in lysosomes, while 25-(OH) vitamin D3 is converted into 1,25-(OH)2 vitamin D3 and resecreted into the circulation (Fig 1) [11]. In the normal kidney, VDBP is reabsorbed by megalin mediated endocytosis and catabolized by proximal tubule epithelial cells reducing the urinary excretion to trace amounts [9].

Acute tubular necrosis (ATN) occurs already during the renal tubular epithelial cell injury when renal blood flow decreases to a level resulting in severe cellular ATP depletion that in turn leads to acute cell injury and dysfunction. Since receptor-mediated uptake of VDBP is energy-consuming, tubular injury is expected to result in urinary VDBP loss [12]. While glomerular filtration rate (GFR) decrease can be diagnosed only hours after renal insult, increased VDBP concentration may be detected as early as ATN occurs.

Administration of iodinated contrast media (CM) increases vasoconstriction and decreases vasodilatation in the renal medulla, leading to hypoxia and even acute tubular necrosis known as contrast-induced nephropathy (CIN) that tends to occur predominantly in diabetics and patients with preexisting renal insufficiency [13].

It was previously shown that urinary VDBP concentration increases with increasing severity of renal damage, and responded to renoprotective therapy [14]. Whether urine VDBP is related to acute tubulointerstitial damage and long term prognosis of the kidney injury has not specifically been addressed so far. We investigated weather urinary VDBP concentration change within the 48 hours after contrast media exposure are a potential predictor of adverse events such as need for dialysis, doubling of serum creatinine, unplanned emergency rehospitalization or death during three months of follow-up and development of contrast media induced nephropathy (CIN). We also compared urinary VDBP and VDBP/urinary creatinine ratio (VDBP/uCr) with established tubular injury markers such as Kidney Injury Molecule-1 (KIM-1) and KIM-1/urinary creatinine ratio (KIM-1/uCr) in patients with high risk for developing CIN.

## Methods

### 2.1 Study design

A prospective cohort of 314 consecutive patients underwent coronary angiography between January 2010 and December 2011 in the Department of Cardiology of the Charité –Universitätsmedizin Berlin. This study was specifically approved by the review board of our institution and by the Ethics Commission of the Charité—Universitätsmedizin Berlin. Each participant of the study has signed an informed consent form prior to involvement into the study. The informed consent form was approved by the Ethics Commission of the Charité—Universitätsmedizin Berlin. The study was conducted according to the Declaration of Helsinki, the European Guidelines on Good Clinical Practice, and relevant national and regional authority requirements and ethics committees. Informed consent was obtained from each participant prior to involvement into the study

#### 2.1.1 Inclusion criteria

Consecutive patients with a high risk-profile of developing contrast induced renal failure, i.e. patients with plasma creatinine of at least 1.1 mg/dl but no dialysis need or patients with preexisting diabetes mellitus independently of plasma creatinine level were enrolled into the study. Inclusion criteria were based on Mehran contrast nephropathy risk score [15].

#### 2.1.2 Exclusion criteria

Patients with end-stage renal disease as well as patients who did not sign an informative consent were excluded from the study.

### 2.2 Course of the study

**Fig 2:**
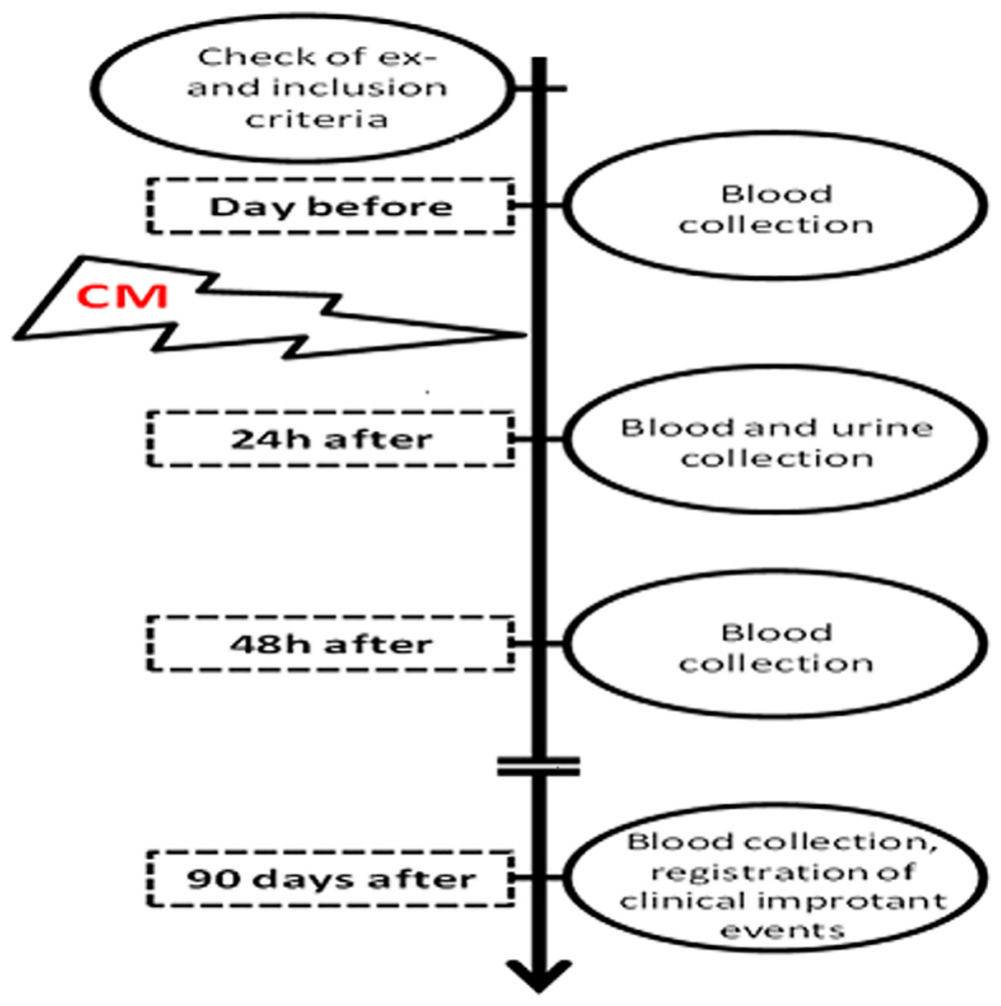
Course of the study.

After enrollment into the study, patients underwent blood- and urine sampling for obtaining basal values. After that, paraclinical examination with contrast media was performed. In the present study, only water-soluble, non-ionic, monomeric, low-osmolar, iodine-based contrast agent Iobitridol was used in a concentration of 350 mg Jod/ml (XENETIX^®^ 350, Guerbet GmbH, Sulzbach/Taunus, Germany). Further, blood- and urine samples were obtained 24, 48 hrs and finally 3 months after contrast agent infusion (Fig 2).

### 2.3 Sample treatment and measurement

The samples were frozen at -80°C the very same day. Before freezing, blood samples were centrifuged 5 minutes with 3000 rotates per minutes and only the plasma was frozen. Creatinine was measured according to the method of Jaffé. GFR was estimated according to the modification of diet in renal disease (MDRD) formula.

Human VDBP was measured with a commercially available sandwich ELISA (Quantikine ELISA No. DVDBPO, R&D Systems, Inc. Minneapolis, USA), according to the manufacturer’s instructions, see: https://resources.rndsystems.com/pdfs/datasheets/dvdbp0.pdf.

For the measurement of Kidney Injury Molecule-1 (KIM-1) in urine, KIM-1 ELISA TEST KIT for the detection of KIM-1 in human (BioAssay Works^®^, L.L.C., Ijamsville, USA) was used according to manufacturer instruction. Kim-1 antigen detection levels in urine greater than 800 pg/ml were realized with a dose response relationship covering a three-log range.

### 2.4 Study endpoints

Study endpoints were death, initiation of dialysis, doubling of serum creatinine, non-elective hospitalization and CIN during the 3 months follow-up. Additionally, incidences of major adverse renal events (MARE) was assessed. CIN was defined as an increase of creatinine of 25% or 0.5 mg/dl from the baseline within 48 hours [16]. MARE was defined as an occurrence of death, initiation of dialysis or a doubling of the creatinine at follow-up.

### 2.5 Statistical analysis

The statistical analysis was made using SPSS 20 (IBM^®^ SPSS^®^ Statistics IBM Cooperation, Armonk, USA). Differences among the biomarkers were estimated using the two-way analysis of variance (ANOVA). Predictive values of the biomarkers were assessed using a logistic regression. Unless otherwise specified, all data are presented as Mean ± Standard Deviation (SD). For all analyses a p < 0.05 was considered statistically significant.

## Results

### 3.1 Patients characteristics

A total of 314 consecutive patients that underwent coronary angiography (239 (76.1%) men and 75 (23.9%) women) with a mean age of 68.89 ± 9.69 years and a body mass index (BMI) of 28.99 ± 5.44 kg/m^2^ were enrolled into the study. 169 (53.8%) patients were previously diagnosed with diabetes mellitus, 81 (25.8%) suffered congestive heart failure and 86 (27.4%) had an anemia (Table 1). The mean volume of injected contrast medium was 112.33 ± 55.24 ml. The means of urinary VDBP and KIM-1 of the entire cohort prior to contrast media exposure were12.80 ± 3515.23 ng/ml and 0.161 ± 0.19 ng/ml respectively. In addition, VDBP/urinary Creatinine (VDBP/uCr) and KIM-1/urinary Creatinine (KIM-1/uCr) ratio as well as GFR were calculated, and were at baseline 1.72 ± 1008.42; 0.026 ± 0.02 and 64.06 ± 21.05 ml/min/1.73 m^2^ respectively (Table 2). The New York Heart Association functional classification (NYHA) [17] was used to grade the severity of functional limitations in a patient with heart failure. NYHA class III/IV was defined as congestive heart failure. 65 patients had a NYHA class III and 16 patients had a NYHA class IV. Anemia was defined as hematocrit of less than 0.36 l/l for females und less than 0.39 l/l for males.

**Table 1:** Patients characteristics at baseline. CM: contrast media, VDBP: vitamin D binding protein, KIM-1: kidney injury molecule 1, uCr: urinary creatinine, CIN: contrast induced nephropathy, GFR: glomerulary filtration rate estimated with the MDRD formula, ME: mean, M: mean, SD: standard deviation,*—p<0.05 is statistically significant.

Patients characteristics	N	314
Female/male	N (%)	75/239 (23.9/76.1)*
Age (ME±SD)	Years	68.89 (±9.69)
Body mass index (ME±SD)	kg/m^2^	28.99 (±5.44)
CM-volume (ME±SD)	Ml	112.33 (±55.24)
Baseline plasma creatinine (ME±SD)	mg/dl	1.24 (±0.43)
Baseline VDBP (ME±SD)	ng/ml	12.80 (±3515.23)
Baseline KIM-1 (ME±SD)	ng/ml	0.161 (±0.19)
Baseline VDBP/uCr (M±SD)	ng/ml/mmol/l	1.72 (±1008.42)
Baseline KIM-1/uCr (M±SD)	ng/ml/mmol/l	0.026 (±0.02)
Baseline GFR (ME±SD)	ml/min/1.73m^2^	64.06 (±21.05)
Diabetes mellitus	N (%)	169 (53.8)
Congestive heart failure	N (%)	81 (25.8)
Anemia	N (%)	86 (27.4)
Smoking	N (%)	199 (63.4)
Essential hypertension	N (%)	278 (88.8)
Obesity: No/BMI 25-30/ BMI 30–35/ BMI 35–40 / BMI > 40	N (%)	77/116/77/32/12 (24.5/36.9/24.6/10.2/3.8)

**Table 2:** Time to death and causes of death during the follow up.

Patient ID	Days from the inclusion into the study to death	Cause of death
**19**	7	Unknown
**29**	90	Bradyarrhythmia with asystole
**42**	84	Respiratory failure
**124**	79	Acute decompensated heart failure
**133**	26	Sepsis
**149**	95	Sudden cardiac death
**164**	70	Sepsis and infective endocarditis
**276**	48	Acute pulmonary embolism with acute decompensated heart failure

### 3.2 Correlation between VDBP and KIM-1 and the study endpoints (Tables 2, 3, 4 and 5)

**Table 3:** Urinary concentration of VDBP and KIM-1 24 hrs after CM injection comparison in patients with and without complications. VDBP: vitamin D binding protein, KIM-1: kidney injury molecule 1, CIN: contrast induced nephropathy, MARE: major adverse renal event, GFR: glomerulary filtration rate estimated with the MDRD formula, N: number of patients, M: mean, SD: standard deviation, p: significance according to MANOVA, p<0.05 –is statistically significant.

		VDBP [ng/ml]			KIM-1 [ng/ml]	
	M	SD	p	M	SD	p
Whole cohort	132.23	340.05		0.161	0.19	
CIN						
No	134.91	355.71	0.668	0.23	0.195	0.795
Yes	169.41	335.72		0.22	0.119	
Death						
Alive	121.41	324.45	0.04	0.24	0.198	0.004
Dead	522.01	521.86		0.31	0.23	
Dialysis						
No	113.06	299.61	<0.001	0.23	0.19	0.041
Yes	613.07	700.45		0.36	0.35	
Non-elective hospitalization						
No	102.81	262.81	0.001	0.24	0.187	0.362
Yes	291.77	573.39		0.27	0.256	
MARE						
No	112.08	302	<0.001	0.18	0.175	0.009
Yes	506.16	624.61		0.32	0.321	

**Table 4:** VDBP/uCr, KIM-1/uCr 24 hrs after CM injection and baseline creatinine comparison in patients with and without complications. VDBP: vitamin D binding protein, KIM-1: kidney injury molecule 1, uCr: urinary creatinine, CIN: contrast induced nephropathy, MARE: major adverse renal event, GFR: glomerulary filtration rate estimated with the MDRD formula, N: number of patients, M: mean, SD: standard deviation, p: significance according to MANOVA, p<0.05 –is statistically significant.

	VDBP/uCr			KIM-1/uCr			Baseline Creatinine [mg/dl]		
	M	SD	p	M	SD	p	M	SD	P
Whole cohort	22.97	75.98		0.03	0.022		1.24	0.43	
CIN									
No	24.18	81.09	0.895	0.03	0.024	0.993	1.24	0.39	0.221
Yes	26.56	58.7		0.03	0.016		1.13	0.36	
Death									
Alive	18.62	61.09	<0.001	0.03	0.022	0.016	1.22	0.39	<0.001
Dead	139.57	222.29		0.05	0.039		1.86	0.98	
Dialysis									
No	16.02	48.05	<0.001	0.03	0.02	<0.001	1.2	0.34	<0.001
Yes	169	244.52		0.06	0.052		2.26	0.95	
Non-elective hospitalization									
No	16.07	54.23	0.002	0.03	0.021	0.009	1.21	0.37	0.013
Yes	54.01	130.36		0.04	0.031		1.39	0.67	

**Table 5:** Logistic regression analyses MARE independent variables. VDBP: vitamin D binding protein, KIM-1: kidney injury molecule 1, p<0.05 –is statistically significant.

Variables	B	SE	Wald	95% CI		*P*
				Lower	Upper	
**VDBP**	0.001	0.001	5.548	1.000	1.002	0.019
**KIM-1**	2.273	1.109	4.200	1.104	85.419	0.040
**Age**	-0.014	0.029	0.216	0.932	1.044	0.642
**Contrast volume**	-0.002	0.005	0.087	0.988	1.009	0.769
**Anemia**	0.539	0.198	7.373	1.162	2.529	0.007
**Congestive heart failure**	0.154	0.122	1.582	0.918	1.482	0.208
**Diabetes mellitus**	-0.317	0.624	.257	.215	2.475	0.612
**Renal insufficiency**	-0.448	1.140	0.155	0.068	5.960	0.694

Eight patients died during the follow-up time of 90 days. Death occurred at a mean of 74.5 (7–95) days after the study entry. The causes of death were cardiovascular diseases in 4 patients, infections in 2 patients, respiratory failure in 1 patient and other/unknown reasons in 1 patient (Table 2). Mean VDBP levels were significantly lower in survivors (121.41± 324.45 ng/ml) compared to deceased patients (522.01 ± 521.86 ng/ml; p = 0.040) (Table 3). Calculated VDBP/uCr ratio confirmed this difference. Urinary KIM-1 and KIM-1/uCr 24 hrs after CM injection as well as baseline creatinine were significantly higher in non-survivors compared to survivors (Tables 3 and 4).

CIN was diagnosed in 21 patients of our study population. Neither VDBP nor VDBP/uCr 24 hrs after CM injection were predictive for CIN. There was also no difference between KIM-1 and KIM-1/uCr in CIN versus non CIN patients 24 hrs after CM injection (Tables 3 and 4).

11 patients in our cohort had to undergo dialysis during the follow-up period. VDBP (Table 3) as well as VDBP/uCr (Table 4) 24 hrs after CM injection were significant predictors of dialysis need, as their values were significantly higher in patients needed dialysis treatment subsequently (613.07 ± 700.45 ng/ml and 169.00 ± 244.52 ng/ml/mmol/l vs 113.06 ± 299.61 ng/ml and 16.02 ± 48.05 ng/ml/mmol/l respectively). Increased urinary KIM-1 as well as KIM-1/uCr also significantly predicted subsequent dialysis need. It has to be mentioned, that patients that underwent dialysis had significantly higher plasma creatinine at baseline (1.20 ± 0,34 vs. 2.26 ± 0.95 mg/dl).

Cumulative occurrence of the major adverse renal events (MARE) defined as an occurrence of death, initiation of dialysis or a doubling of serum creatinine at follow-up seeing in16 patients of our study population could be predicted by significantly higher levels of VDBP as well as VDBP/uCr as early as 24 hrs after CM injection (506.16 ± 624.61 ng/ml and 125.68 ± 211.62 ng/ml/mmol/l vs 112.08 ± 302.00 ng/ml and 14.99 ± 38.10 ng/ml/mmol/l respectively). Increased urinary KIM-1 and KIM-1/uCr ratio were significant predictors of MARE as well (Tables 3 and 4). Logistic regression analyses MARE independent variables confirmed that VDBP along with KIM-1 and anemia were significant predictors of MARE (Table 5).

In addition, we assessed correlation between non-elective hospitalization during the 90 days of follow-up and urinary VDBD, urinary KIM-1, VDBP/uCr and KIM-1/uCr. Statistical analysis revealed that increased levels of urinary VDBD, VDBP/uCr, urinary KIM-1, KIM-1/uCr 24 hrs after CM injection together with elevated plasma creatinine concentration at baseline were significantly higher in patients needed non-elective hospitalization during the 90 days of follow-up. (Tables 3 and 4).

## Discussion

To our knowledge, this is the first study demonstrating that urinary VDBP and VDBP/uCr are biomarkers predicting dialysis need, death, non-elective hospitalization and MARE up to 90 days after contrast media exposure. With the exception of non-elective hospitalization urinary KIM-1 24 hours after CM exposure was likewise significantly higher in patients who died, needed dialysis or developed MARE during the follow-up of 90 days (Tables 4 and 5). Urinary VDBP and KIM-1, however, were not related to the development of CIN.

Our results are consistent with previous reports on the urinary loss of VDBP in the setting of renal damage in a rat adriamycin-induced nephrotoxicity model [18] as well as in the setting of chronic kidney disease in humans [19,20]. Interestingly, urinary VDBP [14] as well as urinary KIM-1 [21] were associated with interstitial inflammation independently of albuminuria rendering VDBP an even more interesting candidate biomarker.

It was previously shown that urinary VDBP was rising with increasing severity of renal damage, and responded by declining to renoprotective therapy [14]. In addition, urinary VDBP is about 4-fold increased in diabetic patients with normoalbuminuria [22]. These facts suggest that tubulointerstitial damage, considered as the final common pathway towards end-stage renal disease (ESRD), is present at the early asymptomatic stage of chronic kidney disease. Indeed, urinary VDBP was strongly and consistently elevated in rats with adriamycin-induced nephropathy very early in the course of the disease, before pro-fibrotic biomarkers could even be detected [14]. In addition, increased urinary VDBP was strongly associated with markers of tubulointerstitial fibrosis after induction of nephrosis [14].

Increased levels of urinary VDBP were significant predictors of all-cause mortality in our study. To our best knowledge, there are no data on association between urinary VDBP and mortality in the current literature. Nevertheless, in a number of studies, low bioavailable vitamin D plasma concentrations were associated with higher mortality rates [23,24].

The US Food and Drug Administration approved KIM-1 as a one of the urinary biomarkers in a panel for preclinical trials [25]. The peak of urinary KIM-1 is 24–48 hrs after onset of the kidney injury [26,27,28,29]. These data are not in line with our results, which have shown no significant association between increase of urinary KIM-1 and CIN. A number of studies reported even that increased urinary KIM-1 was not significantly associated with acute kidney injury [30,31]. Verbrugge et al. in their study on patients with acute decompensated heart failure found that urinary KIM-1 is not a reliable predictor of persistent renal impairment or all-cause mortality, as it was shown in our study. At the same time, the ratio KIM-1/uCr may be of value for the detection of renal injury, as it was described by Kwon et al. in patients with IgA nephropathy. Normalizing a urinary biomarker concentration to urinary creatinine takes into account differences in urinary flow rate. However, if the assessed biomarker behaves exactly like creatinine in terms of filtration, secretion and reabsorption [32], the normalized level may be affected by differences in urinary creatinine excretion. This needs to be considered when “normalizing” urinary biomakers to urinary creatinine concentrations.

The currently used clinical definitions of CIN are based on the short-term changes of glomerular function after contrast media exposure, since they use changes of GFR surrogate biomarkers such as creatinine to define CIN. These definitions are suitable for both daily clinical work and clinical studies. They, however, ignore alterations in tubular function although the morphological hallmark of acute renal failure in general and CIN in particular are tubular alterations such as tubular necrosis and tubular dilatation [33], Vitamin-D-binding protein is filtered through the glomerulus as a 25-(OH) vitamin D 3/VDBP complex and is uptaken by megalin receptors in the brush border of proximal tubule cells [9,11]. This is a highly specific tubular process. Glomeruli are not involved in it. Thus our findings that urinary VDBP concentrations are a biomarker of major clinical outcomes for 90 days after contrast media exposure but not for CIN as defined by short term alterations of GFR indicate that tubular alterations are much closer related to long term clinical outcome then short term alteration of GFR. The current CIN definitions describes more likely short term alterations of glomerular hemodynamics / glomerular function, whereas tubular alterations are obviously much closer linked to contrast media induced long term clinical consequences. This concept is supported also by the KIM-1 data presented in this study. They likewise predict MARE but not CIN and KIM-1 is also just of tubular origin. Our data also questions the currently used clinical definition of CIN based on short term changes in GFR. It might be more appropriate to use biomarkers describing tubular function to define CIN in clinical settings, since tubular alterations are the most prominent morphological finding in CIN and as indicated by our study, tubular biomarker alterations might be better related to the -more important– 90 day clinical outcome after contrast media exposure (MARE). This hypothesis, however, needs to be tested in larger clinical studies.

## Conclusion

Urinary VDBP and VDBP/uCr are promising novel biomarkers of major contrast induced nephropathy-associated events 90 days after contrast media exposure. and may thus be usefuls tools for diagnostics and treatment of acute renal failure after contrast media exposure.
